# Supplementary material for: Integrated group antenatal and pediatric care in Haiti: A comprehensive care accompaniment model
Source: PLoS One. 2024 Jul 12;19(7):e0300908. doi: 10.1371/journal.pone.0300908 (PMC11244772; doi:10.1371/journal.pone.0300908)
Supplement: S2 File — (DOC) [file pone.0300908.s002.doc]

Interview # 5

Interviewer : Bonswa tout moun mwen se **[identifiable data removed]**, mwen ap travay nan hum, nou te mande ou pou vini jodi a pou kapab ede nou konprann pi bien experyans ou antanke on mun ki f pati pwogram nou rele J9 la, nou pral pozew kesyon sa yo ke nou prepare, paske nou panse ke lidew kapab pemet nou amelyore kalite sevis nou ap bay, pandan nou ap ranfose sa ki te déjà fet déjà yo, ki t fet bien yo, nan etid sa a nou pral pozew yon seri de kesyon e nou ap anrejstre vwa ou, antrevi a ap dire ant 30 a 40 minit konsa, sa nap cherche nan menw se idew, kisaw panse, kidonk pa gen ni bon ni move repons, nan sousi pou nou kapab kenbe sekre, nou ta mandew ou pa bezwen site non ou pandan antrevi sa a, tout infomasyon ke wap bay nou jodi a ap rete sekre, repons ou pap gen yon enpak sou patisipasyon ou nan program J9 la. Pou mwen komanse avek kesyon yo mw ta renmen pou nou ta pale de tet nou, moun ki vle reponn ap leve men yo epi nap reponn, pale de tet nou an jeneral tankou kote wap viv, konbien timoun ou genyen, de kote ou soti, jis pale de ou.

Interviewer: Hello everyone my Name is **[identifiable data removed]**, I’m working for HUM, as a member of the J9 program, we asked you to come today to help us better understand your experience, we will ask you few questions, because we believe that your ideas can allow us to improve the quality of our services, while reinforcing what has already been done. In this study we will ask you a series of questions and we will record your voice. The interview will last about 30 to 40 minutes. So, what we are looking for is your idea, what do you think. So, there is no right or wrong answer. We would like to keep it confidential. We will not mention your name during this interview. All the information you give us today will remain confidential. Your answer will not impact your participation in the J9 program.

To start with the questions, I would like you to tell me about yourselves, those who want to answer will raise their hands and we will answer. In general: I'd like you to tell me about yourselves. Where are you from and how do you get here, OK you can talk.

Mwen soti **[identifiable data removed]**, wout Pou antre mache kana poum vini isit la Mwen vini sou machinn epi Lem rive sou place la Mwen pran moto

I’m from **[identifiable data removed]**, the road to enter Kana’s market, and for me to come here I took a car and once I get to the main square, I took a motorbike taxi.

Interviewer: Pa gen lot moun ki vle reponn? pale de tèt nou e koman , koman nou viv, koman nou fè pou rive isit la? Map tande lot repons na

Interviewer: No one else wants to answer? talk about yourselves and how do you live, how do you get here? I’m waiting for your answers.

Mwen soti **[identifiable data removed]** nan wout nan antre òfelina **[identifiable data removed]** nan, gen defwa m fè ti mache a pye pou m soti sou lari pou m pran moto, lè m pa jwenn moto andedan poum vini HUM

I came from **[identifiable data removed]** near the road to the entrance of **[identifiable data removed]** orphanage, sometimes I have to walk down the street to pick up a motorbike taxi on the main road, when I can't find a motorbike on the side road, in order to come to HUM.

Interviewer: OK. Mwen ta renmen pou lot moun pale de tet yo, pale m de tèt, pale m de nou, pa gen lot moun, ok.

Interviewer: OK. I would love for other people to talk, tell me about yourselves, talk about you, no one else, Ok.

Mwen soti **[identifiable data removed]**, sou antre savanèt e mwen pran moto mete m kafou flande epi mwen pran machin meten sou plas la lè m rive sou plas la mwen pran yo moto anko epi mete m nan lopital la HUM.

I’m from **[identifiable data removed]**, at the entrance to Savanette and I take a motorbike taxi that drop me in Flambe crossroad then I take a car that drop me at the main square then I took another moto taxi that drop me here at HUM.

Interviewer: OK, mèsi bouko. Donk, e kisa ki motive nou pou fè pati de pwogram J9 la?

Interviewer: OK, thanks la lot. So, what motivates you to be part of the J9 program? 2;00

Sa ki motive m se paseke m te renmen gen enfomasyon e paske mwen konnen enfomasyon ka fè m sove tèt mwen e sove pitit mwen lè m pral fè e le fet ke sitou se premye pitit mwen, e paske mwen konnen, moun ki te nan J9 deja yo di m ke yo fè fomasyon pou yo, e pandan mwe ladann vre mwen twouve fomasyon an itil, ici mwen ta ansent e ansent anko mwen tap toujou vini nan J9.

What motivates me is because I would like to get information and because I know that information can save myself and my child, above all it is my first child and because I know people who were in J9 already, they told me that they were educated and while I was in J9, truly I found useful information here. If I’m pregnant again I will always come back to J9.

Interviewer: Mèsi bokou. Pa gen lot moun ki vle reponn?

Interviewer: Thank you very much. No one else wants to answer?

Sa m renmen nan J9, se paske tout mis yo, tout doktè yo, yo mete yo nan po yo pou yo kapab fè w aprann sa yo gen pou yo fè w aprann nan, pou n kapab jere tèt ou pandan ou pote bebe a , lè bebe a fin fèt tou pou kapab jere l. Mwen te santi m byen e Mwen te santi m tap viv tou pAndan m tap swiv fomasyon nan J9 yo.

What I love about J9 is that all the nurses and doctors, feel comfortable to teach us what we can learn, so we can take care of ourselves while we are carrying the baby, and when the baby is born, how to take care of the baby. I felt good while I was attending the J9 trainings.

Sak te enterese m nan J9, lè m te vin enskri nan J9 se pandan mwen te vini fè premye konsiltasyon mis lan ki te nan laboratwa, li di m sa paske se zanmi m li te ye. Mwen pat regrèt deske l te di m sa paske mte inskrit nan J9 nou jwenn anpil bon bagay nan J9 malgre se katriyèm pitit mwen, mwe mem jwen anpil swen yo pran ka nou ; ni le ou vin konsulte nou yo rele w pou konsilte, yo toujou byen resevwa w byen e lè ou fin akouche too, yo toujou byen resevwa ou, e sa fè m kontan pou J9 e pap janm pa, mwen pap janm bliye J9 pou jan yo te pran ka nou.

What interested me… when I enrolled in J9, was when I came to my first prenatal visit, and the nurse who was in the lab told me about it, because she is my friend. I am happy that she told me this because I enrolled in J9 and I have found a lot of great things in J9, even though it’s my fourth child, I have found that they take a lot of care with us. When I come in for a consultation they call me for my checkup, and they always welcome us very well and after I delivered as well, they always receive us. That makes me happy for J9 and I will never forget J9 for the way they took care of us.

Inter: Mèsi, bokou.

Interviewer: Thank you very much 3;45

Mwen menm, se yon zanmi ki tap pale m de J9, epi jan li tap pale de J9 lan m de li a mwen di fok m ta sipoze antre, epi lè m antre mwen pa regret paseske mwe te antre nan J9. J9, fè anpil fomasyon pou nou e gen de bagay mwen pat konnen J9 fè mwen konnen anpil bagay sitou se premye pitit mwen, mwen kontan, mwen kontan e m pap janm regret mwen te antre nan J9.

For me, a friend of mine told me about J9, and the way they talked about J9 told me that I had to enter and then when I signed up, I don’t regret it because J9 do a lot of trainings for us and there are things I didn't know before. J9 helped me to know a lot of things above all it is my first baby. I'm happy, I'm happy and I'll never regret that I joined J9. 4;09

Interviewer: Ok, mèsi. Donk, kiyes ki te refere nou nan J9? koman nou te fè rive nan J9?

Interviewer: Ok, thanks. So, who referred you to J9? How did you find out about J9?

Se pandan ke mwen te vin fè egzamen epi doktè nan laboratwa a te dim ap fè ou enskri ou pap pase mizè lè ou vini lopital.

It was when I came to do a laboratory test and the doctor told me to sign so I won’t have to go through a lot of difficulty (mize) when I come to the hospital.

Sak fè m te rive nan J9, paske se yon zanmi m ki te di m gen J9, sa kounye la a pandan mwen te konsilte mwen te tou vini, mwen te rive nan J9.

The reason how I got into J9 was because a friend told me there is J9 and now during my prenatal consultation, I came and I joined J9.

Panden m te vini konsilte, mte vini le mte vini lopital la mpat sante mpat bien mte vini, mfe konsiltasyon epi li soti positif epi gen yon zanmi mwen lap eksplike’m, li te vin nan konsiltasyon an tou epi li gen yon pwogram la ki bon anpil, epi mwen vini vreman vre, mwen pat regret malgre pou li tou li vini men yo te rele l yo pa jwenn li. Men, mwen pat regret paske mwen te vin nan J9, mwen jwenn anpil enfomasyon, bon enfomasyon pou te pwoteje m pandan mwen ansent la, jiskaske mwen akouche e mwen pa regret sa, e yo fè bon travay e bon travay anpil, yo ba nou bon enfomasyon, mèsi.

When I came to have my prenatal consultation, I wasn’t feeling well. I came and in the consultation the test was positive and I have a friend of mine who explained to me when I came for the consultation that there is a program that is really great. And when I really came, I don’t regret it. I got a lot of information - good information to protect myself while I was pregnant until I delivered and I don’t regret that. They did a very good job and a lot of good work, they gave us good information. Thanks. 5;44

Interviewer: Eske nou prè pou nou refere lot fi ki ap pote yon timoun nan pwogram J9 la?

Interviewer: Are you ready to refer other pregnant women to the J9 program? Explain to me

Wi, mwen prè pou m fè sa e m gentan fè sa e paske gen plizye moun m gentan bay, eksplike jan yo eksplike m nan, jan yo fè fomasyon avè m nan, mwen gentan fè anpil fomasyon avek moun, mwen gentan gen nenpot 2 moun mwen eksplike sa epi a no jou la mwen panse map kontinye fè moun toujou konnen sak gen nan J9 paske mwen renmen J9 anpil, mèsi. 6;20

Yes, I am ready to do that and I have been doing this because I already explained to many people the way they explained to me, about the way that they did the training with me. I have done a lot of training with other people, I have already referred two people and I explained that. To date, I think I will continue to let people know what is in J9, because I love J9 so much, thanks.

Interviewer: Mèsi bokou. Gen yon sel moun ki referee n J9?

Interviewer: Thank you very much. There is only one person who refers people to J9?

Ebyen wi, mwen byen kontan m pre pou m refere moun yo vin nan J9, mwen fè sa deja map kontinye fè sa, paske l enpotans mwen li gen pou mwen, gras ak li menm mwen gen pitit mwen an paske gras ak fomasyon li fe pou nou-a mwen te ka konprann kek siy ke m genyen, mwen te kouri lopital, gras ak fomasyon ke yo fè pou mwen, pitit mwen te fet an bòn sante, mwen menm tou mwen an bòn sante. Donk, tout sa motive m pou m refere moun vini nan J9. 7 ;00

Well yes, I am very happy and I am ready to refer people to come into J9, I have already done so, I will continue to do so because the importance for me – it has for me, thanks to it, me I have my baby and because of the training that (J9) did for us, I understand some signs that I have, I run to the hospital. Thanks to this training that they did for me, my baby was born in good health. Me too, I am in good health. Therefor, all this motivates me to refer other women to come to J9.

Wi, mwen kontan paske mwen te nan pwogram J9 ki fè m gen bebe nan menm, mwen pat gen okenn danje ni bebe m. Mwen toujou di moun se J9 la pou yo toujou vini. Mesi 7;14

Yes, I'm happy because I was in J9 that gave me safely a baby. I don’t have any problems neither does my baby. I always tell people about J9, so, they can also join. Thank you.

Interviewer: Mèsi bokou. Pati sa mwen pral poze nou kek kesyon sou eksperyans ke nou fè nan patisipe nan pwogram J9 la, sou sa ou panse nou ka amelyore pou lot moun ki ap fè pati de pwogram nan, ka jwenn pi bon sèvis? Silvouplè, eske nou ka eksplike m koman e kisa ki te fè nou chwazi aksepte fè pati de pwogram J9 la? 7;45

Interviewer: Thank you very much. In this part, I will ask you some questions about your experience that you had in the J9 program. What do you think we can improve for other people who will participate in J9 to find better service in the future? Please, can you explain how and why you chose to be part of the J9 program?

Bon, Sak fè m te fe aksepte fè pati J9, paske premye pitit mwen, premyeman mwen pat konnen koman pou m te jere ansent lan, e mwen te konnen tout siy ki te… ki pou demontre m ke lè m santim pa byen, le… si… timoun pandan mte ansent lap gen pwoblem se sak fè ke mwen te antre nan J9. 8;26

Well, the reason why I accepted to be part of J9, was because of my first child… first of all, I did not know how to manage the pregnancy, and I didn’t know all the signs that…. that demonstrated that I was not feeling well when... if… the baby while I was pregnant there was a problem that was the reason I joined J9.

Interviewer: Mèsi bokou. Eksplike m kisa n panse, explikem koman nou te kon fe jwen yon sevis parèy, sèvis yo bay nan J9 la ak lot kote, eske sèvis nou jwenn nan J9 la egziste lot kote? e koman eksperyans nou te ye avè l? Eksperyans nou fè ak J9 la eske nou fè eksperyans sa yo lot kote, koman sa te ye avel?

R : Experynas Nan J9 ?

Interviewer : Experyans nou te fel nan J9 lan, eske w te fel yon lot kote e koman li te ye ? 9 ;17

Interviewer: Thank you. Explain to me what you think, explain to me how we knew how to receive a service like this? Did you find services provided like J9 elsewhere, does the service at J9 exist elsewhere? and how was that for you ?

R: Experiences in J9?

I: Experiences we had with J9, did you have that anywhere else and how was it?

Non, mwen pa fè eksperyans nan lot kote paske dabitid lè ou vin konsilte la, ou preske fè jounen an, pou deklase dosye epi, yo voye w fè egzamen pafwa ou konn pa menm reyisi konsilte mem. Men ak J9 se diferan paske gen yon gwoup w se rele ou pafwa yo rele w ou gen randevou, e kan w vini ou veye ou chita, pandan wap fè fomasyon an yo konsilte w ou pa menm pran tan mem. si se 8 tè ou gen randevou, 10 zè, ou avan 10zè ou gentan ale lakay ou. Donc, antanke fanm ansent pafwa ou gen malèz. Donk, avek pwogram J9 la se tous kil fo, se tout sa mwen te bezwen. 10 ;04

No, I did not have experience anywhere else, because usually when you go for a prenatal visit, you spend almost the whole day, to get your chart, then they send you to get lab tests, sometimes you aren’t even able to get seen. But with j9, it is different because there is a group and they call you sometimes, they call you for your appointment and when you come, they have you sit down and while you are learning they give you your prenatal check and you don’t even take a lot of time. If it is 8 O’clock you have an appointment, by 10 O’clock or before 10 O’clock you have left and are heading home. So as a pregnant woman sometimes you are not feeling well (have discomfort) so with the J9 program, it has everything necessary; it is all that I need.

Interviewer: Mèsi bokou.

Interviewer: Thank you very much.

Nou we program J9 la diferan de tout lòt lopital yo kote nou kon al konsilte. Dèfwa menm lè nan… kote nan … lopital prive al konsilt e-w, pou un ten ou pase mizè pou deklase dosye pou w sesi. J9 depwi w enskri ladan yo rele w vin konsilte depwi w rive, yo vini yo konsiltew nòmalman. Pwogram j9 diferan de tout lòt kote mem le li te pou lajan yo te pral konsiltew nan klinik prive. 10 ;34

We see the J9 program different from the rest of the hospital where we have consultations. Sometimes even when… where… the private hospitals even when they give consultations, you pass a lot of time suffering to get your medical chart to be seen. With J9, as soon as you are signed up, they call you to come for your appointment and when you arrive, they see you normally. The J9 program is different than anywhere else; even where they take money for a private clinic visits.

Interviewer: Ok, mèsi bokou. Nan kad pwogram nan nou te benefisye de plizyè sèvis; pa egzanp vizit domisilye, konsiltasyon gwoup pedyatri , gwoup prenatal e konsiltasyon e sikososyal , eske w kapap prezante nou selon eskperyans ou pwen fò pwogram nan, pwogram e… vizit domisilyè a , avèk pwen fèb li kisa nou te renmen nan vizit domisilyè a, e kisa nou pat renmen nan vizit domisilyè? 11;16

Interviewer: Ok, thank you. As part of the program you have benefited several services such as: home visits, pediatric groups, prenatal groups and psychosocial consultations. From your experience can you tell us what is the strong points about this program ; the home visits … and the weak points. What you liked about the home visits and what you did not like about the home visits ?

Ben, Pwen fò la se sa m te renmen ladan l, se ke lè yo vizite m, yo te visitem, yo te gade bebe e lonbrit bebe patko tonbe, e mwen menm pat remake sa, e lè yo te konsilte bebe yo te we ke lomblit la, li pa nòmal e yo te di m an ijans m dwe antre lopital la. La fèt ke, li pat twò bonè, epi yo te di m pou m vini demen maten bonè, lopital. Epi m te vini nan J9 pedyadri avè l, donk sa se yon bon bagay, le fèt ke yo pat vini et petet ke li te ka fè enfeksyon oubyen yon lot bagay anko.

Donc pou pwen négatif, mpa genyen. 11 ;55

Well, the strong points that I liked about it, is that when they came to visit me, they saw the baby and they noticed that the umbilical cord had not fallen off yet. Me myself, I had not noticed that and when they examined the baby, they saw that the umbilical area was not normal and they said to me to urgently go to the hospital. The fact that it was late and they told me to go very early the next morning to the hospital. Then when I came to the J9 pediatrician with her, then it was a really good thing. The fact that if they had not come and perhaps she could have had an infection or even something worse (could have happened). So for me I don’t see any weak points.

Mwen te resevwa yon visit nan , Mwen pa jwenn okenn kritik de J9 lè yo te vizite m lan yo te vizitem nòmalman e m pa jwenn okenn kritik de sa , paske yo te byen pale avèk nou e yo te byen resevwa lakay la e yo te pale nomalman, e yo te konsiltem tou; visit ansent. 12;22

I received a visit. I did not find anything critical to say about J9 when they came to visit me. They visited me normally and I did not find any criticism in that because they spoke well, they talked with us and they were well received in my home and they talked normally. They gave me a checkup as well, a prenatal visit.

Interviewer: E nan konsiltasyon prenatal kisa nou kapab di ki te bon ? E ki pa bon nan konsiltasyon prenatal ? Sa vle di konsiltasyon pandan n ap pote bebe a, le nou vini nan konsilatasyon pandan nou pote bebe-a, kisa nou te renmen kisa nou pa t renmen nan konsiltasyon sa ? 12 ;55

Interviewer: And what was good about the prenatal care? And what was not good about the prenatal care? That is to say, the consultations while you were carrying the baby, when you cam to the prenatal consultations; what did you like and what did you not like about those visits?

Mwen menm sa m te renmen nan konsiltasyon an lè m t ap pote bebe a, se pafwa m kon gen malèz lè m vini, m pa konnen, mpa gen mankma si m malèz se lè m rive, epi yap fè m konnen epwi yo konsilte m byen, epi le’m… bebe … kè bebe t ap bat fò, mpat janm konn sa, e lè m rive nan J9 epi yo Di m sa epi you voye m na… na Triage epi yo fin mesire’m, epi yo retoune’m nan J9 anco, sa’m te renmen, m pa gen kritik pou li. Mèsi. 13;27

For me, what I loved about the prenatal consultations while I was pregnant was that sometimes I had discomforts and I did not know what was going on, if I wasn’t feeling well. It was when I arrived that they told me and then they did my checkup and then when… baby … the babys’ heart was beating very fast and I never knew it and when I arrived in J9, they told me and they sent me to the Women’s Emergency Room and there they measured me and checked everything and they sent me back to J9 again. That I loved. I don’t have anything bad to say about that. Thank you.

Nan konsiltasyon j9 m pa jwenn okenn kritik paske kelke swat le’w vini, kèlkeswat pwoblèm ou genyen menm lè l pa dat randevou w yo resevwa nou nomalman. M pa gen kritik pou sa. 13 ;43

In the prenatal J9 visits, I don’t have anything bad to say , because no matter when you come; whatever your problem you have; even if it is not your prenatal visit date, they receive you normally. I have nothing bad to say about that.

Interviewer: Donk e nan… e… sante…. E… konsiltasyon sante mantal, kisa nou te renmen kisa nou pa t renmen lè nou…. Le nou nan konsiltasyon sante mantal? 14 ;06

Interviewer: What about the mental health consultation? What do you like and what didn’t you like when you had a mental health visit?

(music telephone…)

Pou konsiltasyon sante mantal la, m twouve se Yon… se yon bon Bagay paske lè yon fiy, li ap pote Yon bebe sitou avèk òmon yo, no pafwa ou strese, ou ka gen pwoblèm lakay ou avek anviwònman, donc, le fè ke nou te gen sikològ ki te pale avèk nou donk sa fè ke nou mwens estrese e… pou anviwònman m jere anviwònman byen e si m gen yon pwoblèm too ki ap trakase n, donc nou te gen posiblite pou n te gen Yon sikològ ki te pale avèk nou pou ki te ede nou jere pwoblèm nan, sa ki te ka gen yon efè sou bebe a donk se yon inisyativ ke m renmen anpil, e ki te fè byen pou nou nan J9. 14;56

For the mental health consultations, I found them to be very good, because when a woman is carrying a baby, above all, you have lot of hormones and you may sometimes have stress, you can have a prboem in your house or with your environment. So when we have a psychologist to talk to, then that reduces our stress and… for the environment, I managed my environment well and if I had a problem also that knocked me over, then we had the chance to talk with the psychologist to help us manage the problem. That had an effect on the baby so it is an initiative that I loved so much and it is very good for us in J9.

Interviewer: kòman eksperyans nou nan pwogram j9 la amelyore sante nou ak sante pitit nou? Kòman eskperyans nou, ekspernans nou nan pwogram J9 la, explikem koman li amelyore sante nou ak sante pitit nou?

Interviewer: How does the J9 program improve Your health and your children's health? How was your experience in J9? Explain to me how that improved your health and your baby’s health?

Eksperyans nou nan J9 li amelyore sante nou ak sante pitit nou nan sans ke nan fòmasyon yo fè pou nou donc yo pale nou de gwoup manje ke nou dwe manje, nan aliman nou dwe konsome yo k ap konstwi kò a pwoteje kò a e lot anko, donk, le fè ke nou pran aliman sa yo sa fè ke nou gen Yon bebe ki an bòn sante e nou menm tou nou an bòn sante mem aprem fin akouche. 16;00

Our experiences in J9 has improved our health and our children's health in the sense that the training and education they did for us; they talked to us about the dietary food groups that we need to eat, the different foods that we need to consume (the body builders, the protectors and others) then when we eat these foods, it helps us have a healthy baby and us too, we are in good health, even after we deliver.

Interviewer: Ok, mèsi bokou. Kòman, eksplikem eksperyans-w…. eksplikem koman eksperyans-w, nan J9 kapab aide nou amelyore nan sèvis ke n ap bay nan J9 la, kisa ou te renmen kisa ou pa t renmen nan eksperyans sa ? 16;36

Inter: Ok, thank you. How, explain to me, in your experiences, explain to me how do you think J9 can improve on the services? What do you like and what didn’t you like about the experience?

Sa m te renmen nan J9 lem pa t janm… yo pat janm tedekouraje ansam avèk nou yo te toujou konn bay nou yon bon sèvis, sa m - se pa renmem, pa renmem, non, - me sam te panse, m panse tap reme kòmsi… e…e… on medsen nan sal yo te voye nou akouche-a, m panse te gen Yon grann nan j9 ki te ka pran ka nou plis ki te ka bay nou kek sèvis , antan m ap fè Pitit la se sa m te panse , aprèsa m pa kritike de anyen ankò. 17;20

What I liked about J9 was that they never discouraged us. They always provided us with good services. What I did not like, not… this is what I think; I think I would like if… um… um… a doctor in the hospital room where they send us to deliver; I think that if there was someone (elder) from J9 who could take our cases, it would give us some services. While I was delivering, that was what I was thinking. After that, I don’t criticize them for anything else.

Mwen menm m pa kritike anyen de akouchman nan j9 paske yo toujou di nou, le depwi nou gen yon doulè ki grav, ou yon siy ki pa nòmal, pou nou ale nan J9 la, me yo toujou di nou pa bezwen yo nan J9 se ak lot miss nou pral renkonre ; me yo te toujou fè fòmasyon pou nou yo di nou lè nou rive nan J9, piga nou fè malèleve pou fe certain de bagay ki pa bon, si nou fè yon Bagay ki pa bon se de J9 y ap pale, sa fè m kontan tou le m… te gen doule, jou sa m te gen rankont ak J9 ; le m te vini m te konsilte, me mpap gen doule mpap gen anyen ki fe mal, yo voyem la kay mwen. Le m te retoune akouche, yo te resevwam byen. Lot miss yo, yo toujou pran ka nou tou. Menm lè nou vini la pou n deklase dosye, nap pase mise, me le’w ap akouche yo gen bon doule yo resevwa’m nomalman , yo pa… yo pa… Yo pa derefise… yo pa mal sèvi avè w. Sa fè m kontan tou m… m pa regret dinskrit nan J9 paske lot moun yo te… yo te … resevwa menm jan nan J9. E aprè m te akouche tou, Mwen te jwenn J9 ki te vizite m andedan an. 18 ;20

Me, I’m not criticizing anything about the delivery with J9 because they always told us that as soon as once we feel severe pain, that is not a normal sign, we need to go to J9. But they always told us that we don’t need them in J9, it will be with another nurse that we will encounter. They educated us and told us that when we arrive in J9 even if we encounter not very nice people who do certain things that are not good, if we do something that is not good, they will talk badly about J9. That made me happy when… when I had pain, that day I had a J9 visit; when I went for my visit, I did not have pain or anything hurting so they sent me home. When I came back to deliver, they received me well. The other nurse they all took care of me. Even when I came to get my chart, it can take a long time but when you are delivering they had welcomed me normally. They didn’t… they didn’t turn me away… they did not treat me badly. That made me happy as well. I… I don’t regret signing up for J9 because a lot of people they were… they were received the same in J9. And after I delivered as well, I found the J9 team visited me inside.

Interviewer: Ok Mesi beaucoup. Silvouplè eske w ka dim kisa nou t ap swete ki fèt nan J9 pou rann lòt fiy tankou nou ke gen pou fè pati de pwogram nan… jwenn pi bon sèvis toujou?

Interviewer: Ok thank you. Please, can you tell me what you hope that is happening in J9 to give other women as yourselves, who participate in the program to get better service?

Mwen renmen… tout tout tout… tout sa yo mete pou jinèf pa eksamp apre … ou fin… ou fin fè rive sou 9 mwa w akouche gen J9 pedyadri , men sa m ta swete… gen J9 matènite tou. 19 ;11

 I love … everything everything everything… all that they have put in place for J9, for example … you finish …. You finish getting to 9 months, you deliver, there is J9 Pediatrcs, but what I hope… that there will be a J9 maternity as well.

Interviewer: Mèsi beaucoup. Bon, Mwe Mem mpat la. Mwen ta renmen pou nou ta banm Yon ti esplikasyon sou konsèy ekip jinèf la kon bay nou : eske koman nou aplike konsèy sa yo ? (R : Kosey pou ansent ou pou Bebe ?), wi kelkeswat si se pou bebe… pou bebe, konsèy ke ekip la konn ba nou.20 ;00

Interviewer: Thank you.Well, I was not there. What I would like for you to explain to me some advice that the J9 team gave you : how did you apply the advice? [advice for the pregnant woman or for the baby?] Yes, whichever, if for the baby… for the baby, advice that the team gave you.

Konsèy ekip la konn ba nou sitou lè n ap pot bebe, li te konn toujou di nou konsa pa bwè kafe, pa bwè bagay gazez, bagay ki pou kontrarye nou, pou l pa monte tansyon nou, apresa, pou bebe menm yo dil toujou di’w nan konsa …. : (baby noises) eskise’m wi. Pou bebe menm toujou yo toujou pale asanm avèk nou, yo toujou di nou konsa… a fom… fom siveye bebe yo, toujou gade pou nou wè koman bebe, koman n ap fonksyone avèk bebe yo, mèsi. 20;36

Advice that the team gave us above all was when we were pregnant, they always told us like this: do not to drink coffee, don’t drink soda and things that can disturb us, or that could increase our blood pressure. After that, for the baby, even they would always tell us like this …. (excuse me). For the baby always, they always talked with us, they always told us like this … to be sure.. be sure to watch over the babies, to always look to see how the babies are doing, observe how we are working with the babies. Thank you.

Bon, mwen pa gen lontan mwen akouche, yo toujou… lè nou t ap pote bebe, yo toujou di nou konsa pinga nou joure, pinga nou fache, paske sa yo pap bon pou lè nou pral akouche, menm si yon moun ta fè nou yon bagay pinga nou nan joure, toujou rete nan silans pou nou pa strese, toujou chache kote pou nou relax nou, pou bebe yo toujou di nou, e… yo fè fomasyon pou nou, pou nou gade petet siy danje yo tankou pou pla pye yo, si yo pa jon, yo toujou di nou gade je yo, men yo, si yo pa gen, si yo pa gen kriz, sè tou. 21 ;14

Well, it is not a long time since I delivered, they always… when you are pregnant they always tell us like this : don’t insult people, dont get angry, because those things are not good for when we are going to deliver, even if someone does something, don’t insult them, just stay silent in order to not get stressed. Always find somewhere to relax for the baby they always told us and… they gave us information and training for us to be on the lookout for warning signs such as if the bottoms of the feet, make sure they are not yellow, they always told us to look in the eyes, the hands, make sure they are not having a seizure. That’s all.

Non, non konsey pou pot bebe, Yo toujou di nou depi nou santi yon, yon anòmal, tankou si nap bay san, si anba ti vant nou ap fè nou mal, si nou gen tèt fè mal, do pye anfle, do men anfle, nou dwe veye siy danje yo. Epi tou, yo di nou pou n pran manje ki pou konstwi kò, ki pou pwoteje kò, nou konn pran tout kalite manje nou dwe pran yo, yo ba nou anpil lòt konsèy, yo di pinga nou pale anpil, pinga nou joure, pinga nou strese, paske si nou strese bebe a ap konsa tou. Gen yon sèten de bagay nou pa gen dwa fè, nou jwenn anpil bon konsèy nan J9. 21;56

The advice during pregnancy, they always told us that if we feel anything abnormal, such as if we are bleeding, if our lower abdomen is hurting, if we have a headache, if the tops of our feed or the backs of our hands are swollen, we need to be on the lookout for those danger signs. And also they told us to eat the foods that build the body, that protect the body, we know how to eat all the different food groups and what we need to take. They gave us a lot of advice. They told us not to talk a lot, not to insult, not to stress out, because if we get stressed, the baby will feel it as well. There are some things that we should not do, we received a lot of good advice in J9.

Gras ak fòmasyon yo te fè pou nou, nan siy danje yo youn nan bagay ki te itil mwen, tout te ede m, men sa ki te plis ede m, se siy danje yo. Donk, gras ak fòmasyon yo lè m lakay mwen, pandan m lakay mwen m te wè m ap bay glè san, e manmanm te di m ke bon pa enkyete m se prepare m ap prepare m, pou ko, men le fèt ke yo te aprann mwen ke se siy danje, imedyatman mwen te mete rad sou mwen, mte vini e lè m rive lopital la mal nan triyaj la, epi le yo te konsilte m epi yo di m poko prè paske m te nan faz latans lan, lè yo voyem deyò a, epi m wè map bay san, map bay dlo, gen yon moun ki di m konsa : “ah, ou te bwè twòp dlo”, men mwen le fèt ke J 9 fè fòmasyon pou nou, m te wè se te yon siy danje, e lè m rantre m te di yo map bay dlo, imedyatman yo te metem, yo te banm sewòm, banm kabann. E gras ak fòmasyon yo te fè pou nou, pandan m nan peryòd latans lan m te kase lezo, nan faz latans lan m te kase lezo. Donk, le fèt ke m te genyen fòmasyon sa J 9 te fè pou mwen an, donk m genyen bebe. Men, si m te rete pandan ke m te fin kase lezo, likid amyotik pitit la pat ladann m te ka pèdi bebe, donk avèk fòmasyon sa, li itil nou anpil, nou ka genyen bebe, genyen vi nou tou. 23 ;18

Thanks to the education they gave us, one of the warning signs; one thing that was useful for me and helped me, but I can say that they helped me the most were the warning signs. Well thanks to the trainings, when I was at home while I was at my house I saw that I started to have a streak of blood. And my mother told me that it was good, not to worry, it is my body preparing myself, I am getting ready. But the fact that they taught me that it was a warning sign, immediately, I put clothes on and I came and when I arrived at the hospital, I went to Triage (Women’s Health ER) and when they checked me and they told me I am not yet ready because I was in latent labor, when they sent me outside and then I saw that I was bleeding and broke my water. There was someone who told me that “Oh, you drank too much water”, but me, the fact that J9 educated us, I saw that it was a warning sign and when I went back inside and told them that I broke my water, immediately they put me… they gave me an IV and gave me a bed. Thanks to the training that they did for us, when I was in latent labor, when I broke my water in latent labor. So the fact that I had the education J9 gave me, then I have my baby. But if I stayed when I broke my water, amniotic fluid, there wasn’t any inside and I could have lost the baby. Then with the education it was very useful, we have a healthy baby and we saved our lives.

Interviewer : Mèsi. Bon, silvouplè m ta renmen konnen si nan pati pe nan pwogram J9 la si pat gen yon bagay ki koute nou kob, eksplike m koman nou depanse pou sa?

Interviewer: Thank you. Please, I would like to know if the J9 program cost you any money? How much did you spend?

Non, pwogram J9 la pa koute nou senk kòb. Li pa koute nou kob ditou, ditou, ditou, mèsi. 23 ;49

No, the J9 program does not cost any money. It doesn't cost us any money at all, at all, at all. Thank you.

Pwogram J9 pa koute nou kob. Nou pa enskri pou kob, nou pa bay okenn kob, sèlman nou peye frè moto pou nou vini. Men, nou pa bay okenn lajan pwogram J9 ede li ede nou pito. 24;02

The J9 program does not cost money. We do not register for money, and we do not give any money. We only pay for the taxi motorcycle to come here. But we don't give any money to the J9 program, they rather help us.

Interviewer : Mèsi bokou. Silvouplè, m ta renmen konnen eske sa pran nou tan pou nou vini patisipe nan pwogram J9 la? Eksplike m yon yi kras sou sa. (mpa tande byen) Mwe ta remen konnen eske patisipe nan pwogram J9 lan pran nou anpil tan, pou nou vini patisipe nan pwogram J9 la, eske li pran nou anpil tan ? fon ti eksplikem plis sou sa.24 ;46

Interviewer: Thank you very much. Please, I would like to know… does it take you a long time to get into to the program? Explain to me a little about this. (I did not hear well). I would like to know if participating in the J9 program takes a long time. For us to come participate in the program does it take a long time. Please explain more about this.

(baby crying) Vini nan pwogram J9 la, li pranm anpil tan paske poum vini distans pou m soti kote pou m soti pou m vini an, se toujou Mibalè men se yon seksyon kominal li ye, donk se seksyon gaskòy, m oblije pran moto mete m nan vil la e pran moto mete m lopital la. Donk, li pran yon ti tan tou piti.25;12

(baby crying) To get into the J9 program, it takes a long time because for me to come, the distance for me to leave to come here it’s still Mirebalais but it is a communal section. It’s Gazkòy section, I have to take motorbike taxi to Mirebalais center and from there I take another motorcycle to the hospital. So, it takes some time.

Interviewer : Bon, nap fini. Mwen ta renmen pou nou ta di m kisa nan pwogram J9 la nou ta swete amelyore ? oubyen , m ta ka di nou ki rekòmandasyon nou tap fè pou amelyore pwogram J9 la?

Interviewer: Well, we are about to finish. I would like you to tell me, what would you like to improve in the J9 program? Or rather, what recommendations do you have for us to improve the J9 program?

Rekòmandasyon pam nan se toujou menm. Men, tou dabò kite m di mèsi avèk tout ekip la donk avek ki mete pwogram nan. Gras ak pwogram J9 la, donk tout moun ka konstate kapab gen mwens desè bò kote timoun yo, e mwens moun ki mouri, nan fi ki mouri nan eklanmsi tou, paske eklamsi gen eklanmsi apre ou fin fè akouchman, mwen menm m te ka fè eklanmsi sa, paske lè yo rele m pou m vin nan konsiltasyon apre akouchman an, m pat janm konnen si tansyon m te wo, alòske m te gen yon tansyon 17 pa 15 si m pa twonpe m. donk, se lè m vini yo di m ke tansyon m li nan wotè sa. Sim pat, si yo pat rele m pou konsiltasyon apre akouchman sa ka rive lè m te vin la se te yon eklanmsi, m te ka mouri tou. Donk, tou sa donk sa se aloure a felisite, donk m di mèsi avèk pwogram J9 la. Donk, men rekòmandasyon an se, m ta renmen genyen matènite J9 la, donk lè genyen konsiltasyon prenatal la, e apre akouchman J9 pedyatri men lè moun nan pral akouche gen ekip J9 tou pou akouchman, mèsi. 27 ;09

My recommendation is still the same. But first of all, let me thank the whole team that put the program together. Thanks to the J9 program everyone can notice that there are fewer children dying and fewer women who die from eclampsia as well. Because there is post-partum eclampsia, I could have had that because when they called me to come for the postpartum visit, I never knew that my blood pressure was so high. I had a blood pressure of 17 over 15 if I am not mistaken. So when I came and they told me my blood pressure was that high, if I did not… if they did not call me for my post partum visit, I could have had eclampsia or I could have died. So, I need to congratulate and thank the J9 program. So my recommendation is that I would like them to have a J9 maternity, so that there is prenatal J9 consultation, post-partum J9, Pediatrics J9, but then people can also deliver with the J9 team. Thanks.

Interviewer : M ap di tout moun mèsi dèske nou te pataje avèk nou, eksperyans nou sou pwogram J9 la, e mèsi pou tan ke nou te pase ansanm avèk mwen. Donk, dènye, dènye kesyon ke mwen ta renmen poze nou : Kisa ki te plis make nou nan pwogram nan?

Interviewer: I would like to thank everyone for sharing your stories with us and your experiences with the J9 program, and thank you for the time you passed with me. So, last question that I would like to ask you is what was the highlight of the program?

Ou di sa ki te plis make nou nan pwogram J9, tout bagay yo te make yon mo nan lavi nou, yon eksperyans tout vi nou ki pou ede nou, e non sèlman si m te pal se pap pou pitit sa sèlman, menm jan lè nou ta dwe fè yon lot pitit, pata gen J9 nou menm pou tèt nou nap toujou kenbe tout pwogram J9 te konn di nou, tout sa J9 te konn ba nou ki pou ede nou pou lavi. Nou pa aprann J9 sèlman pou bebe nou pote a, men nou pote nou aprann li pou lavi, J9 make nou pou tout lavi nou, pou tout tan nap pase toujou paske se yon bon bagay li tap fè. 28 ;41

You want to say what meant the most to us in J9: Everything impressed us, and changed our lives, our experiences for the rest of our lives for helping us. And not only for this baby, but the same for when we have another baby , we will always remember all of the advice J9 gave us. Everything we learned from J9 will help us for the rest of our lives. We not only learned from J9 advice for during pregnancy, for the baby but we learned them for life. J9 change our life forever, for all that will happen, because it is a good thing they are doing.

Interviewer : Mèsi bokou

Interviewer: Thank you very much.

Donk, sa ki plis make m, se vre ke fòmasyon yo… e… yo ba nou yo, donc sa yo vrèman enpòtan, yo vrèman ede m. Men, lanmou ki gen anndan J9 la li make m tou paske le fèt ke yo fè nou reyini an gwoup, se kom nou te en fanmi, se kom se sè nou ye. Lè nou komanse akouche nou youn ap mande pou lot, e nou se kom nou te reme akouche, e le m te akouche, m sonje m te jwenn 2… mte jwen 2 manman, apresa vin gen yon twazyèm epi vin gen yon katriyèm se kòmsi se te 4 sè ki te la, se kom nou youn tap epole lot, e kan nou yon ap akouche epi lap gade, lap gade sou kaban ap di w poukwa akouche, lel prale lap di’w, m’sot w akouche, map tan’nou. Epi se kom ap… jis apre nou toujou pale nan téléphone, youn ap mande lot koman bebe ye, koman bebe ye, se kom nou vin yon fanmi. Donk, lanmoun ki gen anndan J9 la, donc mwen renmen sa. 29 ;39

So, what impressed me the most…. It’s true that the training and education they… they gave us was really important, it really help me. But the love that is inside J9, that that impressed me even more because the fact that they brought us together in a group, it was like we were in a family. It was like we are sisters. When we delivered we asked after one another, and we were like we loved delivering and when I gave birth, I remember that I found 2 other mothers, after that there was a third and then there was a fourth. That was like there were four sisters that were there, it was like we were shoulders for one another and when we delivered, we watched over the beds. When it was time to leave they said I saw you deliver, I will wait for you. And then it was like… just after that we talked on the phone, one will ask after the others, how are the babies, how is the baby. It is like we came to be a family. So the love that is inside J9, I love that.

Interviewer : Mèsi boukou. Donk, se te tout dènye kesyon m ap remèsye nou yon fwa ankò, donk mèsi pou patisipasyon nou, ok.

Interviewer: Thank you very much. So that was the last question I thank you again. Thank you for your participation, ok.
