# Supplementary material for: Integrated group antenatal and pediatric care in Haiti: A comprehensive care accompaniment model
Source: PLoS One. 2024 Jul 12;19(7):e0300908. doi: 10.1371/journal.pone.0300908 (PMC11244772; doi:10.1371/journal.pone.0300908)
Supplement: Attachment — Submitted filename: RESPONSE TO REVIEWERS.docx [file pone.0300908.s003.docx]

RESPONSE TO REVIEWERS AND EDITOR

REBUTTAL LETTER

**Journal requirements: (ACADEMIC EDITOR)**

When submitting your revision, we need you to address these additional requirements.

1. Please ensure that your manuscript meets PLOS ONE's style requirements, including those for file naming.

<https://journals.plos.org/plosone/s/file?id=wjVg/PLOSOne_formatting_sample_main_body.pdf> and

<https://journals.plos.org/plosone/s/file?id=ba62/PLOSOne_formatting_sample_title_authors_affiliations.pdf>

**Response: Done**

2. We note that you have indicated that there are restrictions to data sharing for this study. PLOS only allows data to be available upon request if there are legal or ethical restrictions on sharing data publicly. For more information on unacceptable data access restrictions, please see <http://journals.plos.org/plosone/s/data-availability#loc-unacceptable-data-access-restrictions> .

**Response: Done. We received approval to share the data.**

3. Please review your reference list to ensure that it is complete and correct. If you have cited papers that have been retracted, please include the rationale for doing so in the manuscript text, or remove these references and replace them with relevant current references. Any changes to the reference list should be mentioned in the rebuttal letter that accompanies your revised manuscript. If you need to cite a retracted article, indicate the article’s retracted status in the References list and also include a citation and full reference for the retraction notice.

**Response: Done**

**Reviewer comments:**
The authors sought to evaluate the impact of the J9 model of care on perinatal outcomes among women in Haiti by employing mixed methods.
Below are a few comments for authors to consider.

1. It seems there was a significant difference between the **ages of cases and controls** (p-0.001). Was matching done? If not, was it considered in the analysis as a confounder since this could impact on the outcome measure?

**RESPONSE**: Thank you for your comment. Matching was not done as our study was not a randomized control trial. However, we do have a lower proportion of teenagers in the intervention group. Hence, in response to your comments, we controlled for maternal age in our logistic regression . We have updated the results and limitation sections to reflect this.

2. If the mean of ages was chosen as a measure of central tendency, then the standard deviation must be indicated and not the range. Did the data show a normal distribution?

The authors should clarify. If not, the median (IQR) will be preferred to the mean.

**RESPONSE**: Thank you for this comment. The age data were normally distributed, both the Skewness and Kurtosis are between -2 and 2, [Skewness for J9 is 0.15 and Kurtosis is –0.49 and for the Usual care group Skewness is 0.25 and Kurtosis is –0.63]. In response to your comment, we added a statement to the result section stating that the maternal age was normally distributed. We also added the standard deviation into Table 1.

3. Table 2. Authors should ensure all the variables with their respective totals are well tabulated including the responses under each category of the variable for consistency (e.g. Delivery type, preeclampsia/eclampsia, post-partum visit < 6 weeks, etc). This has been done in Table 3.

**RESPONSE**: This has been corrected for all tables.

4. In the qualitative aspect of the study, the authors indicated only one FGD (with 8 participants) was used because of the inability to obtain consent from the other study participants involved in the other FGD undertaken. Was this challenge rather not a limitation in achieving saturation or did it have no impact on the outcome?

**RESPONSE**; Thank you for this comment, we agree with this limitation We revised our limitations section to further address this in the 7^th^ paragraph.

“In terms of the qualitative data gathered and analyzed, as explained above, only one focus group was coded and analyzed; however, we detected ~~saturation of~~ recurrent themes and sub-themes in the data provided by each of the participants and feel that the qualitative data analysis provides a holistic view of the patient experience with J9. Conducting individual and/ or focus groups with additional participants in the future would be important to determine if there are other themes regarding participant experiences with J9.”

5. Language editing is required in the sentence at lines 437-439.
**RESPONSE**: Thank you for this comment. We changed the wording to make it less confusing to: “There is a high prevalence of perinatal anxiety and depression in LMICs with depression affecting one in four women [26,27,28,29]. Perinatal anxiety and depression increases the risk for adverse health outcomes for mother and newborn [30].”
